# Capturing complexity in clinician case-mix: classification system development using GP and physician associate data

**DOI:** 10.3399/bjgpopen18X101277

**Published:** 2018-04-10

**Authors:** Mary Halter, Louise Joly, Simon de Lusignan, Robert L Grant, Heather Gage, Vari M Drennan

**Affiliations:** 1 Associate Professor, Faculty of Health, Social Care & Education, Kingston University & St George's, University of London, London, UK; 2 Research Fellow, Social Care Workforce Unit, King's College London, London, UK; 3 Professor of Primary Care and Clinical Informatics, Department of Clinical and Experimental Medicine, University of Surrey, Guildford, UK; 4 Honorary Research Fellow, Faculty of Health, Social Care & Education, Kingston University & St George's, University of London, London, UK; 5 Professor of Health Economics, School of Economics, University of Surrey, Guildford, UK; 6 Professor of Health Care and Policy Research, Faculty of Health, Social Care & Education, Kingston University & St George's, University of London, London, UK

**Keywords:** classification, methods, case-mix, general practice, physician assistants, physician associate

## Abstract

**Background:**

There are limited case-mix classification systems for primary care settings which are applicable when considering the optimal clinical skill mix to provide services.

**Aim:**

To develop a case-mix classification system (CMCS) and test its impact on analyses of patient outcomes by clinician type, using example data from physician associates’ (PAs) and GPs' consultations with same-day appointment patients.

**Design & setting:**

Secondary analysis of controlled observational data from six general practices employing PAs and six matched practices not employing PAs in England.

**Method:**

Routinely-collected patient consultation records (PA *n* = 932, GP *n* = 1154) were used to design the CMCS (combining problem codes, disease register data, and free text); to describe the case-mix; and to assess impact of statistical adjustment for the CMCS on comparison of outcomes of consultations with PAs and with GPs.

**Results:**

A CMCS was developed by extending a system that only classified 18.6% (213/1147) of the presenting problems in this study's data. The CMCS differentiated the presenting patient’s level of need or complexity as: acute, chronic, minor problem or symptom, prevention, or process of care, applied hierarchically. Combination of patient and consultation-level measures resulted in a higher classification of acuity and complexity for 639 (30.6%) of patient cases in this sample than if using consultation level alone. The CMCS was a key adjustment in modelling the study’s main outcome measure, that is rate of repeat consultation.

**Conclusion:**

This CMCS assisted in classifying the differences in case-mix between professions, thereby allowing fairer assessment of the potential for role substitution and task shifting in primary care, but it requires further validation.

## Introduction

Primary health care has a pivotal role in the delivery of health care internationally^1^ and understanding who consults, for what reasons, is important for epidemiology, research, planning, reporting, and quality improvement, as well as for potential use in funding models,^2^ including decisions about optimal professional skill mix. Increasingly, health professionals other than doctors are being employed to address problems of medical staff shortages and rising patient demand.^3–5^ In the UK, this shift is exemplified in the deployment of physician associates (PAs, known outside the UK as physician assistants) in general practice to provide consultations, mainly seeing patients booked into same-day or urgent appointments,^6^ a role similar that of PAs in the US.^7^ Health service planners and other decision makers require evidence as to the effectiveness, safety, efficiency, and acceptability of substituting for doctors, and any valid comparison requires understanding of case-mix.

The International Classification of Primary Care (ICPC) was developed to address the perceived inadequacies of the International Classification of Diseases (ICD)^8^ in describing primary care practice.^9^ The ICPC is well-established in a minority of countries,^9–11^ its unique feature being its biaxial structure, which includes reason for encounter as well as morbidity. Like other classification systems,^12^ the purpose is to describe patient complexity of morbidity (and, in the case of adjusted clinical groups, predicted health resource use^12^) over a period of time.^9^ Complexity in patients^13^ is a contested concept,^14^ and there are some arguments that certain classification systems, such as the ambulatory care groups (ACGs),^15^ are limited by defining chronicity as the likelihood of recurrence of the diagnosis^16^ and do not translate fully into other cultural settings.^17^ Classification methodologies that identify acuity (the level of severity of an illness and immediacy of requirements for care^18^) and the complexity of the patient as a whole, are rare. Where they are used,^19,20^ greater transparency in the reporting of case definitions is argued for.^21^

De Jong *et al* ^22^ presented a general practice classification system, developed using ICPC coding data extracted from routinely-recorded electronic medical records, clustered into chronic and oncological diseases (using Knottnerus *et al* ^23^), or minor illnesses and acute diseases (using the judgments of five experienced GPs). This approach met the needs of this study conceptually (investigating the substitution of doctors by PAs in same-day or urgent consultations in primary care in England^24^); however, only 18.6% of the condition descriptors in the study data could be mapped to those listed in De Jong’s study as acute or minor.^22^ In addition, those listed by De Jong *et al* as chronic^22^ included conditions that the authors would not have expected to receive such a classification (for example, acute myocardial infarction or suicide attempt). This study was also intended to capture the complexity of the patient in primary care, albeit complexity lacking a single definition,^14,25^ as this has been considered an explanation for high intra-GP variability in patient management.^26^ Although full complexity of clinical practice cannot be reflected in patient-centred risk adjustments,^16^ this study sought to address complexity (defined as elements being entwined or interwoven into one system)^27^ by combining the acuity of the patient condition at the urgent appointment with any comorbid chronic conditions into one patient classification descriptor.

In the US, PAs have a long history and there are large numbers working in primary care.^28^ The role has been more recently introduced into the UK^29^ and other countries.^30–33^ While this introduction has largely been in isolated developments,^30^ this is changing rapidly.^34^ Numerous studies describe the types of patient consultations that PAs undertake in primary care but presentation of clearly comparative data on case-mix is less common.^35^ In this study, a picture emerges of either selected consultations for PAs,^36^ or of doctors attending more chronic^37,38^ or more serious problems^39^ while PAs attend more unspecified conditions.^40,41^ In this research^24,42^ it was therefore anticipated that there would be difference in the case-mix of PAs and GPs,^6^ and that this could confound the analyses.^43^

This study reports how a revised classification system was built that addressed complexity in the general practice patient case data, and how the impact of this on the analyses of outcomes of care delivered by GPs and PAs was tested (reported elsewhere^24,42^). The overall aim was to develop a method to describe what clinicians do in a way that would allow different types of clinicians to be meaningfully compared.

## Method

- **Design**: CMCS development and testing, using example patient data from a controlled observational study.
- **Setting:** Data were collected from 12 volunteer general practices in England, six employing a PA and six not, matched by health economy and patient population size, during two study reference periods in 2011–2012.^24,42^
- **Participant data**: The study included 2086 routinely-collected electronic consultation records of patients who attended a same-day PA (*n *= 932) or GP (*n *= 1154) appointment (the predominant appointment type for PAs at the time^25^) in the study periods. Data were extracted on condition, as documented in free text and/or in a coding system (the majority of the practices used Read Codes^44^). Data were also extracted on the number and type of chronic conditions the patient had, as recorded on a disease register (that is, the UK primary care pay-for-performance [P4P] Quality and Outcomes Framework^45^ ). 
- **Intervention**: The intervention involved the development of the primary care CMCS and the testing of its impact on the analysis of outcomes of consultation with different types of clinicians, in this case PAs and GPs. The definition of terms used is shown in **Box 1**.

**Box 1.** Definitions of terms used within the case-mix classification system (CMCS)

Term	Definition used in the CMCS
**Acuity**	Severity, intensity, and immediacy of care required for a presenting condition in a patient.
**Complexity**	Interaction between existing conditions and the condition the patient presents with.
**Hierarchy**	Ranking by relative status of acuity and complexity.
**Categorisation**	Assigning each presenting condition to a category defined by acuity.
**Classification**	Assigning each person into a hierarchical system according to their condition category or categories.
**Case-mix**	The mix of patients, according to their classification.

### Development of a primary care CMCS

Concepts and their definitions were discussed by the research team, and the concept of hierarchy evolved during in-person and email discussion, continued until consensus was reached. Following this, each patient condition in the dataset was searched for by LJ and MH in the lists De Jong *et al* classified as acute or minor,^22^ and those with a direct match to a diagnosis were assigned De Jong *et al*’s categorisation. All remaining conditions in the dataset were assigned to one of De Jong *et al*’s categories, or to categories further defined by the GP author (SdeL); these decisions were based on the range of patient conditions in the data, and SdeL’s experience in chairing international primary care medical informatics groups and multiple publications on this issue.^46–50^ As a second stage, each patient consultation — frequently containing more than one condition in one category, or conditions in different categories — was placed into a hierarchy that allowed the patient to move up the hierarchy of complexity in the presence of one presenting condition in a higher (more acute) category, or the registration of a patient on a P4P^45^ chronic conditions register.

### Testing the impact of the CMCS on analyses of PA and GP outcomes

The amount of movement was described from the categorisation of a first condition to the overall classification of that patient. Analyses of the odds or rate ratio for the primary outcome (rate of reconsultation for the same problem within 14 days) and process outcomes (advice given, and numbers of diagnostic tests, referrals, prescriptions, and procedures) for PAs against the reference of GPs were carried out under three alternative adjustment models: case-mix only; other predictor or confounding variables only; and case-mix with other predictors or confounders (the fully adjusted model). The impact of each of these adjustments was assessed using generalised estimating equation models (GEE).^51^ The correlation matrix was set to be exchangeable; that is, patients were assumed to have some shared characteristics within each practice, whether from demographics or organisation of the practice. Cases with missing data on any one of the variables entered into each of the GEEs were excluded for analysis of that outcome. Analyses were carried out using IBM SPSS (version 22).

The study’s anticipated outcomes were a developed primary care case-mix classification system and an assessment of the impact of this on analysis of processes and outcomes of care of different clinicians, using the example of PAs and GPs.

## Results

### The resultant structure of the primary care CMCS

The classification developed contained five categories: acute, chronic, minor problem or symptom, prevention, and process of care. These were considered in a hierarchy of medical acuity, starting with acute through to a process of care, as shown in Box 2.

**Box 2.** Primary care case-mix and complexity index

Case-mix and complexity index classification	Definition of the classification	Definition of the classification for each patient
**Acute**	1. Recent or rapid onset and of short duration (<4 weeks), such as acute pain. 2. Serious, sometimes requiring immediate intervention, such as acute abdomen.^52^	≥1 problem the patient presents with is classified as acute by de Jong e*t al* ^22^ or by current authors.
**Chronic**	(synonym: longterm) As used in ICPC relating to an illness or disability of ≥6 months duration.^52^	Patient has no acute problems; AND patient has a chronic condition, as recorded on a disease register, (with any presenting problem) OR ≥1 problem the patient presents with is classified as chronic.
**Minor problem or symptom**	Minor acute illnesses include some of the commonest problems presented in general practice, such as upper respiratory tract infections or skin rashes.^53^	Patient has no acute problems, no record on a disease register, no chronic problems, AND ≥1 problem the patient presents with is classified as a minor problem or symptom by de Jong *et al* ^22^ or the current authors.
**Prevention**	Action to avoid occurrence or development of a health problem and/or its complications. Can be divided into four categories: Primary prevention: action taken to avoid or remove the cause of a health problem in an individual or a population before it arises. Includes health promotion and specific protection, such as immunisation. Secondary prevention: action taken to detect a health problem at an early stage in an individual or a population, thereby facilitating cure, or reducing or preventing it spreading, or reducing or preventing its long-term effects (for example, methods, screening, case finding, and early diagnosis). Tertiary prevention: action taken to reduce the chronic effects of a health problem in an individual or a population by minimising the functional impairment consequent to the acute or chronic health problem (for example, prevent complications) diabetes). Includes rehabilitation. Quaternary prevention: action taken to identify patient at risk of overmedicalisation, to protect them from new medical invasion, and to suggest to them interventions, which are ethically acceptable.^52^	Patient has no acute problems, no record on a disease register, no chronic problems, no minor problems or symptoms, AND ≥1 problem the patient presents with is classified as prevention.
**Process of care**	(synonym: procedure) In medical care, constitutes the actions undertaken by a physician.^52^	Patient has no acute problems, no record on a disease register, no chronic problems, no minor symptoms or problems, and no prevention problems, AND ≥1 problem the patient presents with is classified as a process of care.

### Placing the example presenting condition data into a category

The 2086 patients in the study presented with 1147 condition descriptors. Examples of the categories applied are as follows: an ‘alcohol withdrawal-induced seizure’ or ‘hyperemesis gravidarum’ was classified as acute; an ‘asthma review’ or ‘multiple sclerosis’ was classified as chronic; ‘productive cough’ or ‘diarrhoea and vomiting’ as a minor problem or symptom; ‘HIV screening’ or ‘smoking cessation’ as prevention; and ‘removal of suture from skin’ or ‘solicitor’s report’ as a process of care. A full listing of condition descriptors and their categorisation is available from the authors on request.

### Placing the example patients’ data into the CMCS: changes to classification level associated with the measure of patient complexity

Three-quarters (*n* = 1548) of the patients in the study presented with only one condition; 38.3% of these (*n* = 593) had a condition co-terminous with one described as chronic. Of the quarter (*n* = 538) of the patients presenting with >1 condition, 397 (73.8%) of these presented with ≥2 conditions that all received the same individual category (for example, four minor problems or symptoms presentations), but 133 (33.5%) of these patients also presented with a chronic condition. The remainder (*n* = 141) of those presenting with >1 condition did so with problems that received differing categorisations; of these 91 (64.5%) also presented with a chronic condition. The classification afforded, according to the category or categories of the patient’s urgent and chronic conditions in combination, is shown in Table 1.

**Table 1.** Listing of presenting condition classification and disease register combinations forming patients’ case-mix classification

Patient case-mix classification	Condition category	Disease register	Total*, n*	GP, *n*	PA, *n*	Change in hierarchy from condition category or categories to patient-level classification
Acute	Acute	No	31	19	12	No
	Acute	Yes	27	17	10	
	Acute + chronic	No	1	0	1	
	Acute + chronic	Yes	3	2	1	
	Acute + chronic + minor	No	0	0	0	
	Acute + chronic + minor	Yes	5	3	2	
	Acute + minor	No	13	9	4	
	Acute + minor	Yes	15	11	4	
Subtotal acute			95	61	34	
Chronic	Chronic	No	23	14	9	No
	Chronic	Yes	69	62	7	
	Chronic + minor	No	19	10	9	
	Chronic + minor	Yes	56	43	13	
	Chronic + process	Yes	1	1	0	
	Chronic + minor + process	Yes	2	2	0	
	Minor	Yes	608	355	253	Yes (n = 639)
	Minor + process	Yes	9	4	9	
	Prevention	Yes	3	3	0	
	Process	Yes	19	10	9	
Subtotal chronic			809			
Minor	Minor	No	1146	571	575	No
	Minor + process	No	14	5	9	
	Minor + prevention	No	3	0	3	
Subtotal minor			1165			
Prevention	Prevention	No	4	3	1	No
Process	Process	No	13	7	6	No

To exemplify the categories in combination, the patient who presented for ‘stiff neck’ (categorised as a minor problem or symptom) and ‘anxiety states’ (categorised as acute) was classified in complexity as acute. The patient presenting with ‘dyspepsia’ (categorised as a minor problem or symptom) and also recorded as being on the chronic kidney disease register was classified as chronic. This patient-level classification resulted in a higher classification of acuity and complexity for 639 (30.6%) of the patient cases in this sample; of these cases, 58.2% (*n* = 372) had consulted a GP and 41.8% (*n* = 267) a PA. Data for each patient case are available from the authors on request.

### Testing the impact of the classification system on analyses of the processes and outcomes of care by different types of clinician, using the PA and GP example

A small number of cases had missing data on variables added to the analysis; the sample size used for each analysis was therefore *n* = 929 for PA consultation and *n* = 1148 for GP consultation.

The case-mix of the two clinician types differed in this study, and the CMCS was therefore used in all analyses to ensure that case-mix differences were adjusted for in any comparison.^24,42^ The results presented in Table 2 show three sets of analyses: unadjusted; adjusted solely for CMCS; and adjusted for CMCS and other relevant variables (age, Index of Multiple Deprivation [IMD], and number of visits in the previous 3 months). The analyses use the three main classifications: acute, chronic and minor, excluding process and procedure classifications due to small numbers involved.

**Table 2.** Crude, CMCS-adjusted, other variable-adjusted and CMCS and other variable-adjusted odds ratios or rate ratios of process and outcome measure differences between PAs and GPs^24^

Process or outcome measure (as defined elsewhere^24^)	Crude/univariate finding		OR or RR (95% CI)				
	GP (reference group)	PA	OR or RR	Unadjusted	Adjusted for CMCS only	Adjusted for other variables of relevance^a^, but not CMCS	Fully adjusted: CMCS and other variables of relevance
Processes	% cases						
General advice	22.9	51.4	OR	3.56 (2.58 to 4.29)	3.58 (1.82 to 7.01)	3.30 (1.68 to 6.47)	3.30 (1.69 to 6.46)
Advice on medication management	12.6	17.1	OR	1.43 (1.12 to 1.82)	^b^	1.62 (1.05 to 2.49)	1.72 (1.08 to 2.72)
Advice on over the counter medication	9.5	20.5	OR	2.45 (1.92 to 3.18)	^ b^	6.63 (0.56 to 4.69)	1.74 (0.62 to 4.89)
	**Mean per case**						
Number of diagnostic tests	0.34	0.36	RR	1.06 (0.82 to 1.38)	1.11 (0.86 to 1.43)	1.07 (0.90 to 1.29)	1.08 (0.89 to 1.30)
Number of referrals	0.11	0.9	RR	0.84 (0.57 to 1.22)	^ b^	0.94 (0.63 to 1.41)	0.95 (0.63 to 1.43)
Number of prescriptions	0.78	0.89	RR	1.18 (0.86 to 1.58)	1.17 (0.88 to 1.55)	1.16 (0.86 to 1.56)	1.16 (0.87 to 1.53)
Number of procedures	0.1	0.1	RR	0.76 (0.32 to 1.84)	^ c^	^c^	^c^
Outcome							
Reconsultation for the same or a linked problem at the practice or urgent care facility within 14 days of the index consultation	0.29	0.32	RR	1.03 (0.75 to 1.43)	1.09 (0.77 to 1.54)	1.12 (0.81 to 1.54)	1.25 (0.91 to 1.72)

^a^With an independent association with outcome (see full report for detail).^24^ ^b^Could not be estimated in SPSS. ^c^Numbers too small for further adjustment. CMCS = case-mix classification system. OR = odds ratio. RR = risk ratio.

Adjusting for CMCS changed differences between the two groups of clinicians notably from the crude estimates, as in the case of 'advice on medication management'. Other measures, such as the 'number of referrals', were changed little by CMCS but notably by other demographic variables. For the primary outcome of re-consultation rate, the adjustment for CMCS alone resulted in only a small change in the estimate, while the larger change was associated with a combination of the CMCS and other variables (in this case: age, IMD, and number of visits in the previous 3 months).

## Discussion

### Summary

This study developed a classification aiming to address complexity in general practice by combining severity and medical immediacy of the urgent condition with information about any chronic conditions. A greater proportion of cases for one clinician type (GP rather than PA cases here) were classified as complex as a result of this, and making allowance for this complexity (rather than for urgent condition alone) resulted in some changes in the estimates of difference between the two groups.^52,53^ The classification system is replicable, using the detailed list of conditions included in each category, the hierarchy, and examples from the full dataset.

### Strengths and limitations

The disease register measure selected for this study as a measure of comorbidity was part of a P4P scheme.^45^ While P4P is potentially effective in changing physician behaviour, it may lead to some gaming and focus on some activities at the expense of others.^54,55^ It also takes a single disease-based approach.^56^ This study may therefore have failed to fully represent complexity. It also may not have been able to distinguish levels of acuity within, for example, conditions such as abdominal pain. However, a measure of complexity has been achieved beyond that previously used in research on this topic, and this has been done using not only system codes but also clinicians’ free text descriptors. The authors acknowledge that, in using the judgments of one family physician (as opposed to a panel of five by De Jong *et al* ^22^) issues of potential differences of clinical opinion, or of bias, have not been addressed.

### Comparison with existing literature

This study set out to both develop a classification system and test its impact on analysis, and therefore has two sets of interpretations. It is recognised that any classification system is reductionist and use of the biopsychosocial model including the patient’s reason for encounter may be preferable.^56^ Classification systems are also critiqued for failing to capture complexity and the undifferentiated symptoms encountered commonly in primary care;^56^ to have struggled with the concept of symptoms at all;^57^ and to change identity through the fact of classification.^58^ Research using any coded information from primary care databases, if used without free text, can miss cases of relevance.^59^ In using free text to inform this classification system, alongside coded information, this study aims to lay a foundation for more accurate comparison of the workload among the primary care workforce.

In terms of the application of the classification system to analyses of groups of clinicians, similar PA and GP case-mix difference has been reported elsewhere.^37–40^ However, comparison is limited by the implicit rather than explicit use of terms such as ‘minor’ or ‘chronic’ in previously published studies, and the lack of adjustment for these case-mix differences in analyses of outcome.

### Implications for research

Task-shifting includes the creation of new professional groups, whereby tasks are shifted from workers with more general training to workers with specific training for a particular task.^60^ However, PAs are trained as generalists rather than specialists,^61^ and may be seen as an example of where task shifting can be a promising policy option to increase the productive efficiency of the delivery of healthcare services.^60^ However, a records-based analysis is required to measure the impact of skill-mix on task-shifting in primary care, and such analyses should include careful statistical adjustment based on explicit rather than implicit definitions of case-mix classification. The system proposed in this study requires further testing, refinement, and validation of the classification in other studies.

In conclusion, this study demonstrated the development of a classification of complexity as well as the difference that applying such classification to analyses makes to results of comparisons of different professionals working in the same system. In particular, the classification allows analyses to account for greater complexity in the patients seen by the GPs. Policymakers, workforce planners, and employers of PAs, or any other clinician substitutes, will need to consider these differences both when considering task allocation and when evaluating efficiency (including cost) and effectiveness. Researchers should be encouraged to consider further developing and evaluating the classification in other studies.

## References

[1] World Health Organization (2008). Primary care: putting people first. World Health Report 2008: Primary Health Care — Now More than Ever.

[2] National Casemix and Classification Centre, University of Woolongong (2017). Comments on the document “Activity based funding for Australian public hospitals: towards a pricing framework”. http://ahsri.uow.edu.au/content/groups/public/@web/@chsd/documents/doc/uow119700.pdf.

[3] The Joint Commission, with support from Aramark (2017). *Health care at the crossroads: guiding principles for the development of the hospital of the future*.. https://www.jointcommission.org/assets/1/18/Hosptal_Future.pdf.

[4] Health and Education National Strategic Exchange (2017). Review of Medical and Dental School Intakes in England. https://www.gov.uk/government/uploads/system/uploads/attachment_data/file/213236/medical-and-dental-school-intakes.pdf.

[5] The Connexion (2017). Stats reveal truth on GP shortage. http://www.connexionfrance.com/statistics-reveal-truth-on-shortage-of-french-doctors-11594-news-article.html.

[6] Drennan V, Levenson R, Halter M Physician assistants in English general practice: a qualitative study of employers' viewpoints. *J Health Serv Res Policy*.

[7] Morgan PA, Abbott DH, McNeil RB (2012). Characteristics of primary care office visits to nurse practitioners, physician assistants and physicians in United States Veterans Health Administration facilities, 2005 to 2010: a retrospective cross-sectional analysis. Hum Resour Health.

[8] World Health Organization (2003). International Classification of Primary Care (ICPC-2)..

[9] Soler JK, Okkes I, Wood M (2008). The coming of age of ICPC: celebrating the 21st birthday of the International Classification of Primary Care. Fam Pract.

[10] Australian Institute of Health and Welfare (2004). *Australia's Health 2004*.

[11] de Lusignan S (2005). Codes, classifications, terminologies and nomenclatures: definition, development and application in practice. Inform Prim Care.

[12] Starfield B, Lemke K (2017). Complexity of patient illness versus complexity of patient visits. http://www.annfammed.org/content/8/4/291.long/reply#annalsfm_el_13780.

[13] Safford MM, Allison JJ, Kiefe CI (2007). Patient complexity: more than comorbidity. the vector model of complexity. J Gen Intern Med.

[14] Green LA (2010). The implications of measuring complexity. Ann Fam Med.

[15] Weiner JP, Starfield BH, Lieberman RN (1992). Johns Hopkins Ambulatory Care Groups (ACGs). A case-mix system for UR, QA and capitation adjustment. HMO Pract.

[16] Katerndahl DA, Wood R, Jaén CR (2010). A method for estimating relative complexity of ambulatory care. Ann Fam Med.

[17] Halling A, Fridh G, Ovhed I (2006). Validating the Johns Hopkins ACG Case-Mix System of the elderly in Swedish primary health care. BMC Public Health.

[18] Brennan CW, Daly BJ (2009). Patient acuity: a concept analysis. J Adv Nurs.

[19] Everett CM, Thorpe CT, Palta M (2013). Division of primary care services between physicians, physician assistants, and nurse practitioners for older patients with diabetes. Med Care Res Rev.

[20] Parkerson GR, Michener JL, Yarnall KS (1997). Duke Case-Mix System (DUMIX) for ambulatory health care. J Clin Epidemiol.

[21] Bhattarai N, Charlton J, Rudisill C (2012). Coding, recording and incidence of different forms of coronary heart disease in primary care. PLoS ONE.

[22] De Jong J, Visser MR, Wieringa-de Waard M (2011). Exploring differences in patient mix in a cohort of GP trainees and their trainers. BMJ Open.

[23] Knottnerus JA, Metsemakers J, Höppener P (1992). Chronic illness in the community and the concept of 'social prevalence'. Fam Pract.

[24] Drennan VM, Halter M, Brearley S (2014). Investigating the contribution of physician assistants to primary care in England: a mixed-methods study. Health Services and Delivery Research.

[25] Peek CJ, Baird MA, Coleman E (2009). Primary care for patient complexity, not only disease. Fam Syst Health.

[26] Rethans JJ, Saebu L (1997). Do general practitioners act consistently in real practice when they meet the same patient twice? Examination of intradoctor variation using standardised (simulated) patients. BMJ.

[27] Sturmberg JP, Martin CM, Katerndahl DA (2017). It is complicated! Misunderstanding the complexities of 'complex'. J Eval Clin Pract.

[28] American Academy of Physician Assistants (2017). Physician assistant census report: results from the 2010 AAPA Census. http://www.aapa.org/workarea/downloadasset.aspx?id=1454.

[29] Drennan VM, Chattopadhyay K, Halter M (2012). Physician assistants in English primary care teams: a survey. J Interprof Care.

[30] Frossard LA, Liebich G, Hooker RS (2008). Introducing physician assistants into new roles: international experiences. Med J Aust.

[31] Jones IW, Hooker RS (2011). Physician assistants in Canada: update on health policy initiatives. Can Fam Physician.

[32] Nederlandse Associatie Physician Assistants [Dutch Association of Physician Assistants] (2017). English Information. http://www.napa.nl/kennisbank/english/ .

[33] O'Connor TM, Hooker RS (2007). Extending rural and remote medicine with a new type of health worker: physician assistants. Aust J Rural Health.

[34] Gallagher J, BBC News (2017). NHS plans rapid expansion of 'doctor's assistant' jobs. http://www.bbc.co.uk/news/health-28896625.

[35] Halter M, Drennan V, Chattopadhyay K (2013). The contribution of physician assistants in primary care: a systematic review. BMC Health Serv Res.

[36] Fethke C, Ekwo E, Daniels M (1979). Management practices: task allocation between physicians and physician assistants. J Ambul Care Manage.

[37] Nelson EC, Johnson KG, Jacobs AR (1977). Impact of medex on physician activities: redistribution of physician time after incorporating a medex into the practice. J Fam Pract.

[38] Grzybicki DM, Sullivan PJ, Oppy JM (2002). The economic benefit for family/general medicine practices employing physician assistants. Am J Manag Care.

[39] Duttera MJ, Harlan WR (1978). Evaluation of physician assistants in rural primary care. Arch Intern Med.

[40] Parle JV, Ross NM, Doe WF (2006). The medical care practitioner: developing a physician assistant equivalent for the United Kingdom. Med J Aust.

[41] Simkens ABM, van Baar ME, van Balen FAM (2009). The Physician Assistant in General Practice in the Netherlands. J Physician Assist Educ.

[42] Drennan V, Halter M, Joly L (2015). Physicians Assistants and General Practitioners: a comparison of same day appointment consultations. Br J Gen Pract.

[43] Dean BB, Lam J, Natoli JL (2009). Review: use of electronic medical records for health outcomes research: a literature review. Med Care Res Rev.

[44] NHS Digital (2017). Read Codes. https://digital.nhs.uk/article/1104/Read-Codes.

[45] NHS Employers (2017). 2014/15 General Medical Services (GMS) Contract Quality and Outcomes Framework (QOF): guidance for GMS contract 2014/15. http://www.nhsemployers.org/~/media/Employers/Publications/2014%2015%20QOF%20guidance%20stakeholders.pdf.

[46] de Lusignan S (2005). Codes, classifications, terminologies and nomenclatures: definition, development and application in practice. Inform Prim Care.

[47] de Lusignan S, van Weel C (2006). The use of routinely collected computer data for research in primary care: opportunities and challenges. Fam Pract.

[48] de Lusignan S, Liaw ST, Michalakidis G (2011). Defining datasets and creating data dictionaries for quality improvement and research in chronic disease using routinely collected data: an ontology-driven approach. Inform Prim Care.

[49] de Lusignan S, Chan T, Jones S (2011). Large complex terminologies: more coding choice, but harder to find data — reflections on introduction of SNOMED CT (systematized nomenclature of medicine — clinical terms) as an NHS standard. Inform Prim Care.

[50] de Lusignan S (2015). In this issue: ontologies a key concept in informatics and key for open definitions of cases, exposures, and outcome measures. J Innov Health Inform.

[51] Snijders TAB, Bosker RJ (2012). Multilevel analysis: an introduction to basic and advanced multilevel modeling.

[52] Bentzen N (2003). Preface. Wonca Dictionary for General/Family Practice.

[53] Jones R, White P, Armstrong D (Managing acute illness). http://www.kingsfund.org.uk/sites/files/kf/field/field_document/managing-acute-illness-gp-inquiry-research-paper-mar11.pdf.

[54] Dixon A, Khachatryan A, Wallace A (2011). The Quality and Outcomes Framework (QOF): does it reduce health inequalities?.

[55] Smith PC, Mossialos E, Papanicolas I (2017). Performance measurement for health system improvement. Experiences, challenges and prospects. Copenhagen: World Health Organization 2008 and World Health Organization, on behalf of the European Observatory on Health Systems and Policies. http://www.who.int/management/district/performance/PerformanceMeasurementHealthSystemImprovement2.pdf.

[56] Gillam S (2010). Should the quality and outcomes framework be abolished? Yes. BMJ.

[57] Soler JK, Okkes I (2012). Reasons for encounter and symptom diagnoses: a superior description of patients' problems in contrast to medically unexplained symptoms (MUS). Fam Pract.

[58] Armstrong D (2011). Diagnosis and nosology in primary care. Soc Sci Med.

[59] Ford E, Nicholson A, Koeling R (2013). Optimising the use of electronic health records to estimate the incidence of rheumatoid arthritis in primary care: what information is hidden in free text?. BMC Med Res Methodol.

[60] Fulton BD, Scheffler RM, Sparkes SP (2011). Health workforce skill mix and task shifting in low income countries: a review of recent evidence. Hum Resour Health.

[61] Miles DL, Rushing WA (1976). A study of physicians' assistants in a rural setting. Med Care.

